# METABOLOMIC PROFILE OF DIFFERENT DIETARY PATTERNS AND THEIR ASSOCIATION WITH FRAILTY

**DOI:** 10.1093/geroni/igac059.1234

**Published:** 2022-12-20

**Authors:** Toshiko Tanaka, Sameera Talegawkar, Yichen Jin, Qu Tian, Eleanor Simonsick, Luigi Ferrucci

**Affiliations:** National Institute on Aging, Baltimore, Maryland, United States; Department of Exercise and Nutrition Sciences, Milken Institute School of Public Health, The George Washington University, Washington, District of Columbia, United States; Nutrition Sciences, Milken Institute School of Public Health, The George Washington University, Washington, District of Columbia, United States; National Institutes on Aging, Baltimore, Maryland, United States; National Institute on Aging, Baltimore, Maryland, United States; National Institute on Aging, Baltimore, Maryland, United States

## Abstract

Diet quality is an important behavioral determinant of healthy aging, however the mechanisms underlying the protective effect of diet are not fully understood. To address this question, we explored whether plasma metabolites mediate the relationship between diet and frailty index (FI) in the Baltimore Longitudinal Study of Aging. Adherence to different dietary patterns was evaluated using the Mediterranean diet score (MDS), Mediterranean-DASH Diet Intervention for Neurodegenerative Delay (MIND), and Alternate Healthy Eating Index 2010 (AHEI). Higher adherence to the dietary patterns was associated with lower FI. There were 127 plasma metabolites that were associated with all three dietary patterns including lipids, fatty acids, ceramides, amino acids, and bile acids. Metabolomic signatures of diet mediated 42%, 70%, and 61% of the association between MDS, MIND, and AHEI with FI, respectively. Overall, we found common metabolomic biomarkers of dietary patterns that explain part of the relationship between diet and frailty.

